# Association of Dll4/Notch and HIF-1a -VEGF Signaling in the Angiogenesis of Missed Abortion

**DOI:** 10.1371/journal.pone.0070667

**Published:** 2013-08-09

**Authors:** Yan Fang, Shuang Yu, Yuyan Ma, Ping Sun, Daoxin Ma, Chunyan Ji, Beihua Kong

**Affiliations:** 1 Department of Obstetrics and Gynecology, Qilu Hospital, Shandong University, Ji’nan, Shandong, China; 2 Department of Hematology, Qilu Hospital, Shandong University, Ji’nan, Shandong, China; Baylor College of Medicine, United States of America

## Abstract

**Background:**

Dll4/Notch and HIF-1a-VEGF have been shown to play an important role during angiogenesis, but there are no data about their roles and association in missed abortion. In this study, we investigated the association of Dll4/Notch and HIF-1a-VEGF signaling in missed abortion.

**Methods:**

Women with missed abortion (n = 27) and healthy controls (n = 26) were included in the study. Real-time Reverse Transcription-PCR Analyses (RT-PCR) was used to analyze the mRNA levels of Dll4/Notch and HIF-1a-VEGF signaling molecules. The protein level for Dll4 was measured by immunohistochemistry.

**Results:**

Compared with induced abortion, the expression of VEGF was statistically reduced while the level of VEGFR1 and Notch1 was significantly up-regulated in missed abortion. Though other molecules (VEGFR2 and Dll4) were marginally higher in missed abortion, no statistical difference was observed. The expression of HIF-1a was significantly up-regulated, and close negatively correlated with VEGF in missed abortion. Both in induced abortion and missed abortion, Dll4 was positively correlated with Notch1.

**Conclusions:**

The early pregnancy is in a hypoxic environment, this may encourage the angiogenesis, but severe hypoxic may inhibit the angiogenesis. Aberrant Dll4/Notch and HIF-1a-VEGF signaling may have a role in missed abortion.

## Introduction

Missed abortion is a pregnancy in which there is a fetal demise (usually for a number of weeks) without outside intervention, but the uterine activity is absent to expel the products of conception before 20 weeks gestation [1]. Multiple etiologic factors, including parental chromosomal abnormalities, uterine abnormalities, hereditary thrombophilia, endocrinological disorders, immunological factors, infections, nutritional and environmental factors, have been identified for missed abortion [2], [3]. However, why pregnancy loss takes place remains unknown. Abnormal angiogenesis is considered as one of the most important reasons of missed abortion.

During development, primary vasculogenesis serves as the template from which a higher order of branching network is generated by the process defined as angiogenesis. Among the great number of genes involved in regulation of vasculogenesis and angiogenesis, significant defects of the development of the vascular system and death of embryos were caused by deletion of VEGF or Delta-like ligand 4 (Dll4) [4], [5].

Vascular endothelial growth factor (VEGF) is believed as the most powerful angiogenesis promoter, VEGF played the significant role in the development and maintenance of the vasculature. VEGF and its receptors are indispensable for the formation of primary vascular network and secondary angiogenesis. Under hypoxic conditions, hypoxia inducible factor 1a (HIF-1a) pathway can release angiogenesis promoters like VEGF [6], [7].

Dll4 is one of the earliest genes expressed in vascular endothelial cells, and is essential for establishment of the vascular endothelial cell fate. Remarkably, the deletion of a single Dll4 allele may result in early embryonic lethality due to the failure to form a functional vasculature [8], [9]. Haploinsufficiency of Dll4, like that of VEGF, leads to embryonic lethality in mice due to vascular defects demonstrating the essential role of Dll4 in angiogenesis during development [8], [10]–[11]. Dll4 is one of the Notch ligands in mammalian cells, and is expressed specifically in the physiological and pathological vasculature [12]. Dll4 and its cognate receptor Notch1 are expressed specifically at sites of vascular development and angiogenesis [13]–[15].

Previous reports demonstrated that Dll4 was a hypoxia-regulated gene, which responded to HIF-1a-VEGF signaling via the hypoxia pathway [16]. Inhibition of the active state of endothelial cells due to stimulation of DLL4/Notch-associated transduction of intracellular signal is evidently caused by lowering of their sensitivity to VEGF [17]. VEGF receptors was direct target gene regulated by Dll4 [18].

Recent studies demonstrated that there may be a feedback loop that links Dll4/Notch and HIF-1a-VEGF in development and angiogenesis. It had also been reported that the up-regulation of Dll4 by VEGF was mediated by both VEGFR1 and VEGFR2. Up to date, Dll4/Notch and HIF-1a-VEGF have been shown to play an important role during angiogenesis, but there are no data about their roles and association in missed abortion. Combining these, we hypothesized that Dll4/Notch signaling might participate in the HIF-1a-VEGF pathway to regulate angiogenesis in missed abortion.

To further investigate the association of Dll4/Notch and HIF-1a-VEGF signaling pathways in missed abortion, we measured the Dll4/Notch and HIF-1a-VEGF pathway molecules and evaluated their clinical relevance. The results showed that DLL4 was a vascular regular involved in pregnancy angiogenesis, and that it was regulated by HIF-1a and VEGF during pregnancy progression under hypoxic conditions.

## Materials and Methods

### Study Population

After approval by institutional review board, 27 women (mean age 28.38, range years 18–41) with missed abortion and 26 women (mean age 27.82, range years 19–40) undergoing surgical termination of normally progressing pregnancies were studied. All study subjects involved in the investigation were recruited following informed consent.

By ultrasound examination, the first-trimester missed abortion was defined as an intact gestational sac lacking any fetal cardiac activity [6 weeks after last menstrual period (LMP)], intrauterine gestational sac with the largest diameter exceeding 10 mm but devoid of yolk sac or an empty gestational sack with a confirmed gestational age of no <6 weeks [1]. All the pregnancies terminated at the first trimester <10 weeks from the LMP. Prior to inclusion in the study, all subjects underwent a standard diagnostic work-up to rule out any verifiable cause of missed abortion. The women were examined using ultrasonography for uterine abnormalities, and blood was drawn for testing for chromosomal abnormalities, immunologic factors (such as positive anticardiolipin antibody and positive antinuclear antibody) and infections, with these analyses resulting in an unexplained etiology.

The control group consisted of 26 women in early pregnancy with a healthy, viable intrauterine fetus and no prior miscarriage. Fetal cardiac activity and gestational age were confirmed by ultrasound. Written informed consent was obtained from all participating subjects. The study design was approved by the Ethical Committee of Shandong University.

Villous samples from the missed-abortion group were collected by curettage or manual vacuum aspiration. Villous samples from the control group were obtained by vacuum aspiration from women undergoing elective abortion at 7–10 weeks of gestation for social reasons. We picked villous tissues under inverted microscope. Each villous sample was divided into two parts: one part was stored at −80°C in aliquots for RNA isolation, and thawed only once to avoid degradation; the other part was stored in 4% formaldehyde at room temperature overnight for immunohistochemistry analysis.

### Real-time Reverse Transcription-PCR Analyses (Real-time RT-PCR)

Total RNA was isolated by Trizol (Invitrogen) according to the manufacturer’s instructions. The quantity of RNA was assessed spectrophotometrically. The OD260/280 of the RNA samples ranged between 1.80 and 2.00. The total RNA was reverse transcribed into cDNA using RevertAid™ First Strand cDNA Synthesis Kit (MBI, Fermentas, USA) in a volume of 20 µl, including 4 µl of 5×reverse transcriptase buffer, 2 µl dNTP (10 mmol/l), 1 µl RNAse inhibitor, 1 µl Reverse Tra transcriptase, 1 µl Oligo (dT) 18 (0.5 µg/µl) and 5 µl total RNA in a thermal cycle. Reverse transcription reaction was done at 42°C for 1 h, followed by duration of 95°C for 5 min.

Real-time PCR was conducted using the Light-Cycler rapid thermal cycler system 2.0 (Roche Diagnostics Ltd, UK) in accordance to the manufacturer's instructions. The PCR mixture consisted of 10 µmol/l of each primer 1 µl, 10 µl Mix SYBR Green 1 (Toyobo) and 500 ng cDNA to a final volume of 20 µl. For negative controls, we used a complete DNA amplification mix in which the target cDNA template was replaced by water. Cycling parameters were the following: denaturation for one cycle at 95°C for 10 s, 45 cycles(temperature transition of 20°C/s) of 95°C for 0 s,58°C for 10 s and 72°C for 10 s and fluorescence reading taken at 72°C, and melting curve analysis with continuous fluorescence reading. The PCR products were analyzed by melt curve analysis and agarose gel electrophoresis to determine product size and to confirm that no by-products were formed. The relative concentrations of the PCR products derived from the target gene were calculated using LightCycler System software. The results were expressed relative to the number of GAPDH transcripts used as an internal control. All experiments were conducted in triplicate. The primers and annealing temperatures used for the amplification of human Dll4/Notch and HIF-1a-VEGF pathway molecules were shown in Table 1.

**Table 1:** Primers and annealing temperatures (temp) for Notch and VEGF pathway molecules.

Genes	Forward (5′to 3′)	Reverse (5′to 3′)	Size(bp)	temp(°C)
Notch1	TCAGCGGGATCCACTGTGAG	ACACAGGCAGGTGAACGAGTTG	104	62
DLL4	CCCTGGCAATGTACTTGTGAT	TGGTGGGTGCAGTAGTTGAG	74	58
VEGF	CCTGGTGGACATCTTCCAGGAGTACC	GAAGCTCATCTCTCCTATGTGCTGGC	196	61
VEGFR1	CTGGACTGACAGCAAACCCAAG	CCACAGCTGGAATGGCAGAA	117	60
VEGFR2	AGCCAGCTCTGGATTTGTGGA	CATGCCCTTAGCCACTTGGAA	133	58
HIF-1a	CATCAGCTATTTGCGTGTGAGGA	AGCAATTCATCTGTGCTTTCATGTC	83	58
GAPDH	GCACCGTCAAGGCTGAGAAC	TGGTGAAGACGCCAGTGGA	138	62

### Immunohistochemistry

Formalin-fixed, paraffin-embedded tissue sections were deparaffined in xylene, rehydrated in grade alcohols, and briefly microwaved in citrate buffer to optimize antigen retrieval. Nonspecific binding sites were blocked with diluted goat serum for 30 minutes at room temperature. Slides were then incubated with rabbit polyclonal primary antibodies raised against human Dll4 (ab7280, Abcam, UK) at 4°C overnight at a 1∶300 dilution. PBS was used for all subsequent washes and for antiserum dilution. After extensive washing (3×5 min) to remove excess antibody the sections were incubated with biotinylated goat anti-rabbit immunoglobulin (Ig) G for 20 min at 37°C and then processed according to the SP kit Zhongshan Co. Ltd, Beijing, China) protocol. Briefly, the slides were stained with DAB (diaminobezidin) for 5 to 10 minutes, and rinsed with PBS for three times. Then, the slides were counterstained with hematoxylin for 2 minutes and observed under microscope. Negative controls for each slide were prepared by substituting the primary antiserum with non-immune IgG. For each experiment, all slides were stained in a single batch and thus received equal staining.

### Statistical Analysis

Statistical analyses were performed using SAS version 9 software. Because of nonnormal distribution or heterogeneity of variance, all data are shown as median (range). Comparison between groups was analyzed by Wilcoxon rank-sum test. Spearman’s test was used for correlation analysis. P-value <0.05 was considered statistically significant.

## Results

### Clinical Relevance of Dll4/Notch and HIF-1a-VEGF Pathway Molecules in Missed Abortion

There was no significant association of the occurrence of missed abortion with the age of women experiencing missed abortion. Correlations between age and Dll4/Notch or HIF-1a-VEGF molecules were analyzed in missed abortion, no statistical difference was found.

### Aberrant Expression Profile of Dll4/Notch and HIF-1a-VEGF Pathway Molecules in Missed Abortion

**Figure 1:**
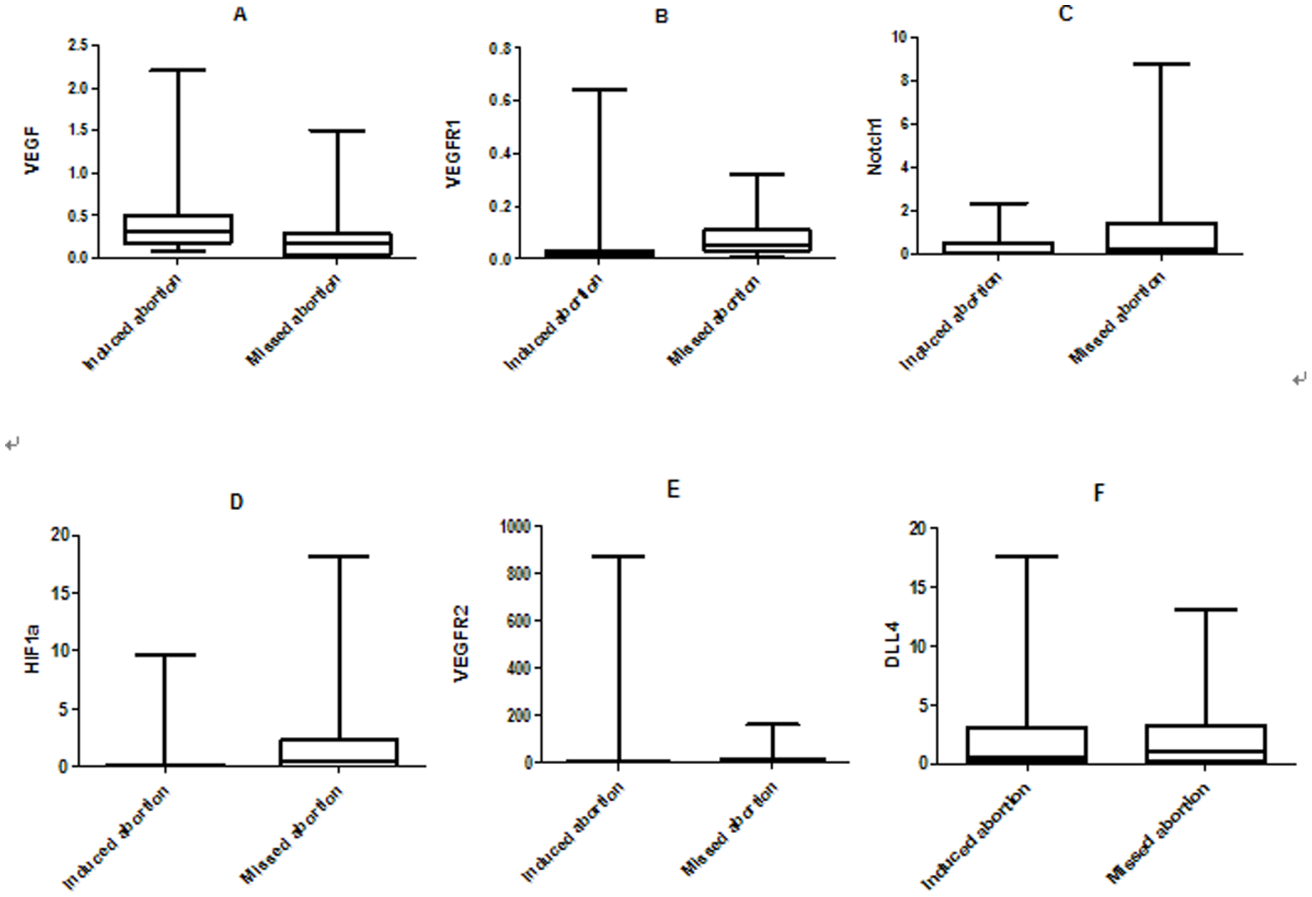
Analysis of Dll4/Notch1 and HIF-1a-VEGF signaling molecules using Real-time RT-PCR. A. VEGF expression was significantly down-regulated in missed abortion compared with induced abortion. B, C, D. VEGFR1, Notch1 and HIF-1a expression were significantly up-regulated in missed abortion compared with induced abortion. E, F. VEGFR2 and Dll4 expressions were higher in missed abortion than in induced abortion, whereas no statistical difference was observed.

To examine whether Dll4/Notch and HIF-1a-VEGF pathway molecules are involved in missed abortion, their expression patterns were investigated using Real-time RT-PCR method. Compared with induced abortion, the expression of VEGF was statistically reduced in missed abortion, while the levels of VEGFR1 and Notch1 were significantly up-regulated in missed abortion. Though other molecules (VEGFR2 and Dll4) were marginally higher in missed abortion, no statistical difference was observed. Besides, the expression of HIF-1a was also significantly up-regulated in missed abortion. (Fig. 1, Table 2).

**Table 2:** VEGF and Notch pathway molecules RNA levels in missed abortion and induced abortion.

molecules	Median(range)		p Value
	Missed abortion	Induced abortion	
VEGF	0.17(0.02∼1.50)	0.305(0.08∼2.21)	0.0136
VEGFR1	0.05(0.006∼0.32)	0.02(0.0021∼0.64)	0.0171
VEGFR2	5.15(0.00000134∼164.00)	0.415(0.0000017∼871.00)	0.4603
Notch1	0.28(0.0036∼8.75)	0.115(0.00537∼2.38)	0.0462
Dll4	1.00(0.01∼13.09)	0.47(0.01∼17.59)	0.5218
HIF-1a	0.39(0.000215∼18.24)	0.00256(0.000000639∼9.69)	0.0318

**Figure 2:**
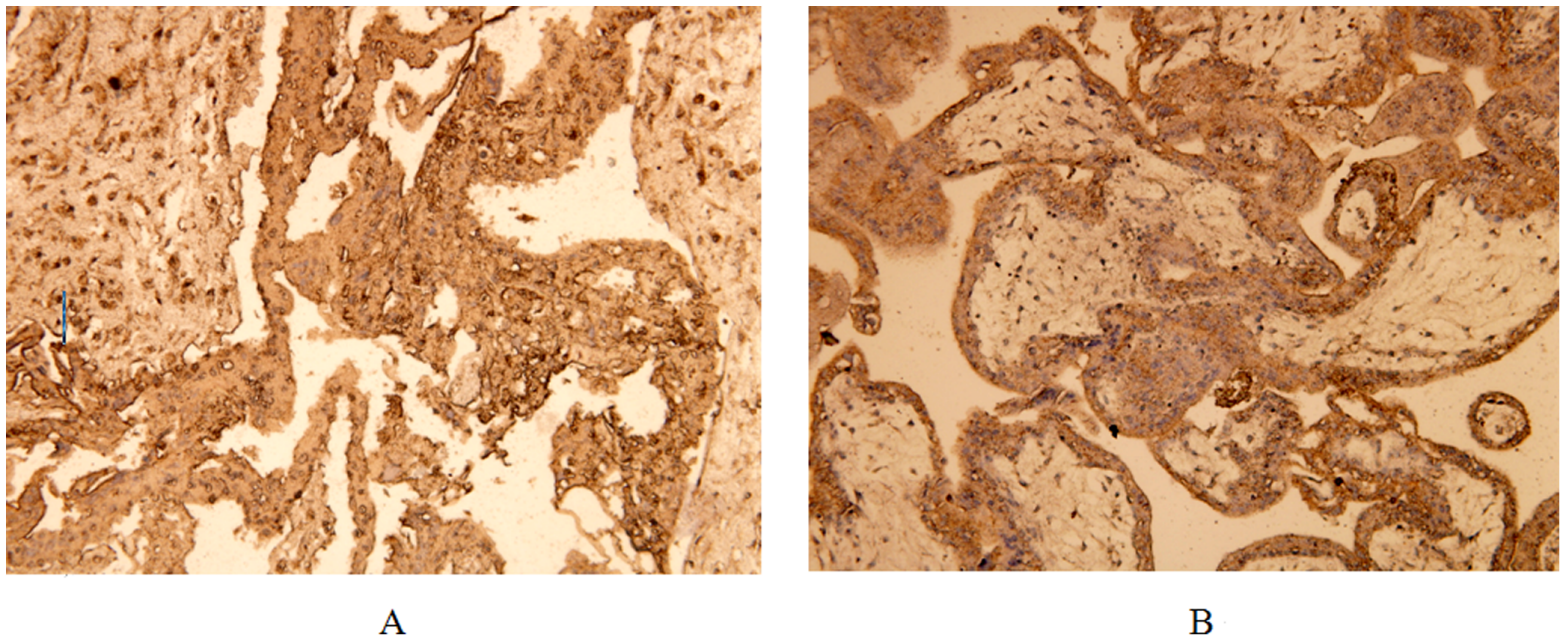
Analysis of Dll4 using immunohistochemistry. The protein expression of Dll4 was up-regulated in missed abortion (A) than that in induced abortion (B).

To verify the results of Real-time RT-PCR, immunohistochemistry was used to detect Dll4. Similarly, the protein expression level of Dll4 was also higher in missed abortion (Fig. 2).

### Correlation Analysis of Dll4/Notch and HIF-1a-VEGF Pathway Molecules in Missed Abortion and Induced Abortion

**Figure 3:**
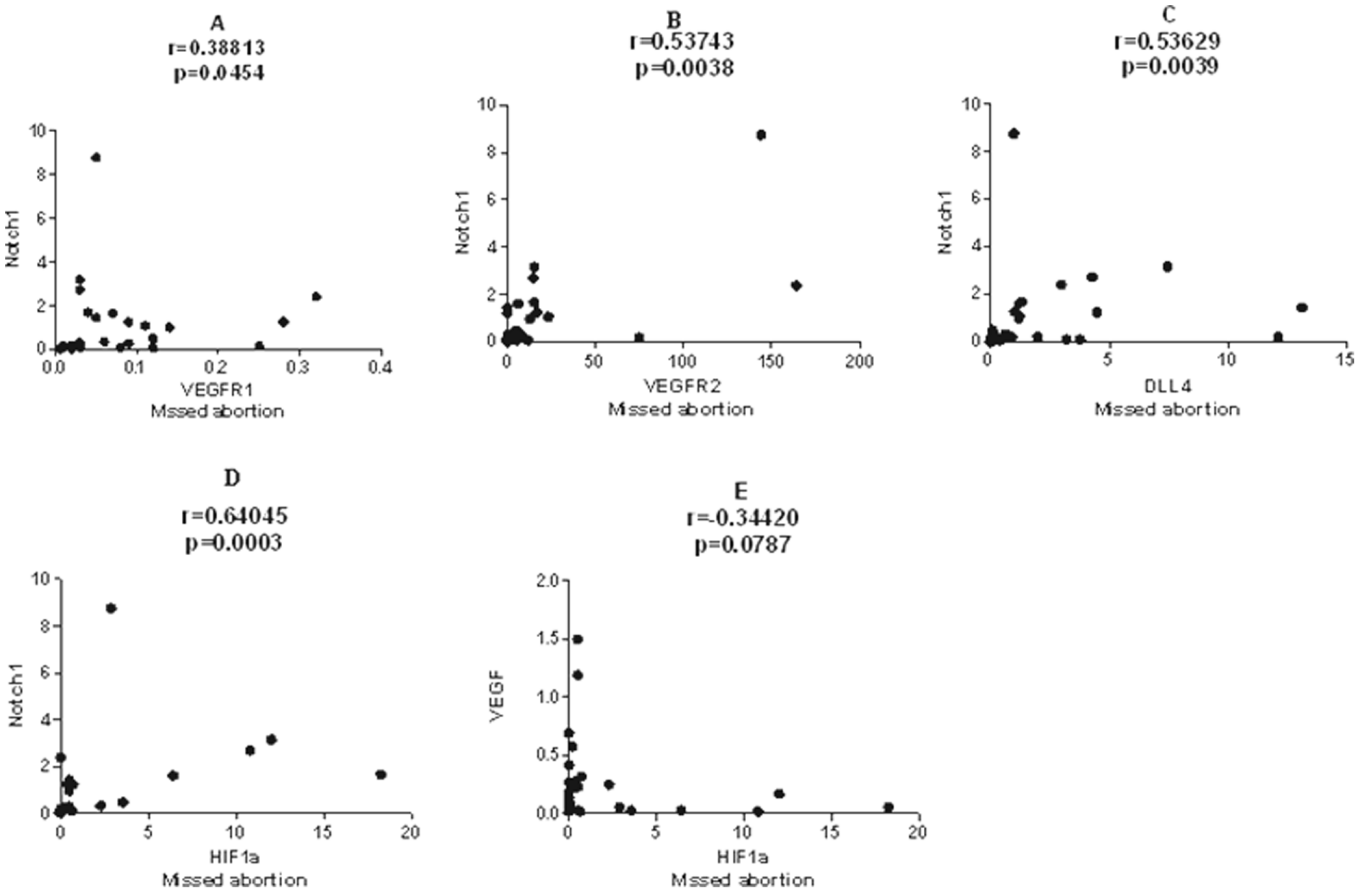
Correlation analysis in missed abortion. A. the expression of VEGFR1 positively correlated with Notch1 in missed abortion; B. VEGFR2 expression positively correlated with Notch1 in missed abortion; C. Dll4 expression positively correlated with Notch1 in missed abortion; D. HIF-1a positively correlated with Notch1 in missed abortion; E. HIF-1a was closely negatively correlated with VEGF in missed abortion.

Spearman correlation coefficients have been calculated between these factors in missed abortion. VEGFR1 was positively correlated with Notch1 (r = 0.38813, p = 0.0454); VEGFR2 was positively correlated with Notch1 (r = 0.53743, p = 0.0038); Dll4 was positively correlated with Notch1 (r = 0.53629, p = 0.0039); And HIF-1a was positively correlated with Notch1 (r = 0.64045, p = 0.0003). Besides, HIF-1a was close negatively correlated with VEGF (r = -0.34420, p = 0.0787) in missed abortion. However, there were no correlations between Dll4 and VEGF/VEGFR1/VEGFR2 in missed abortion. (Fig. 3).

**Figure 4:**
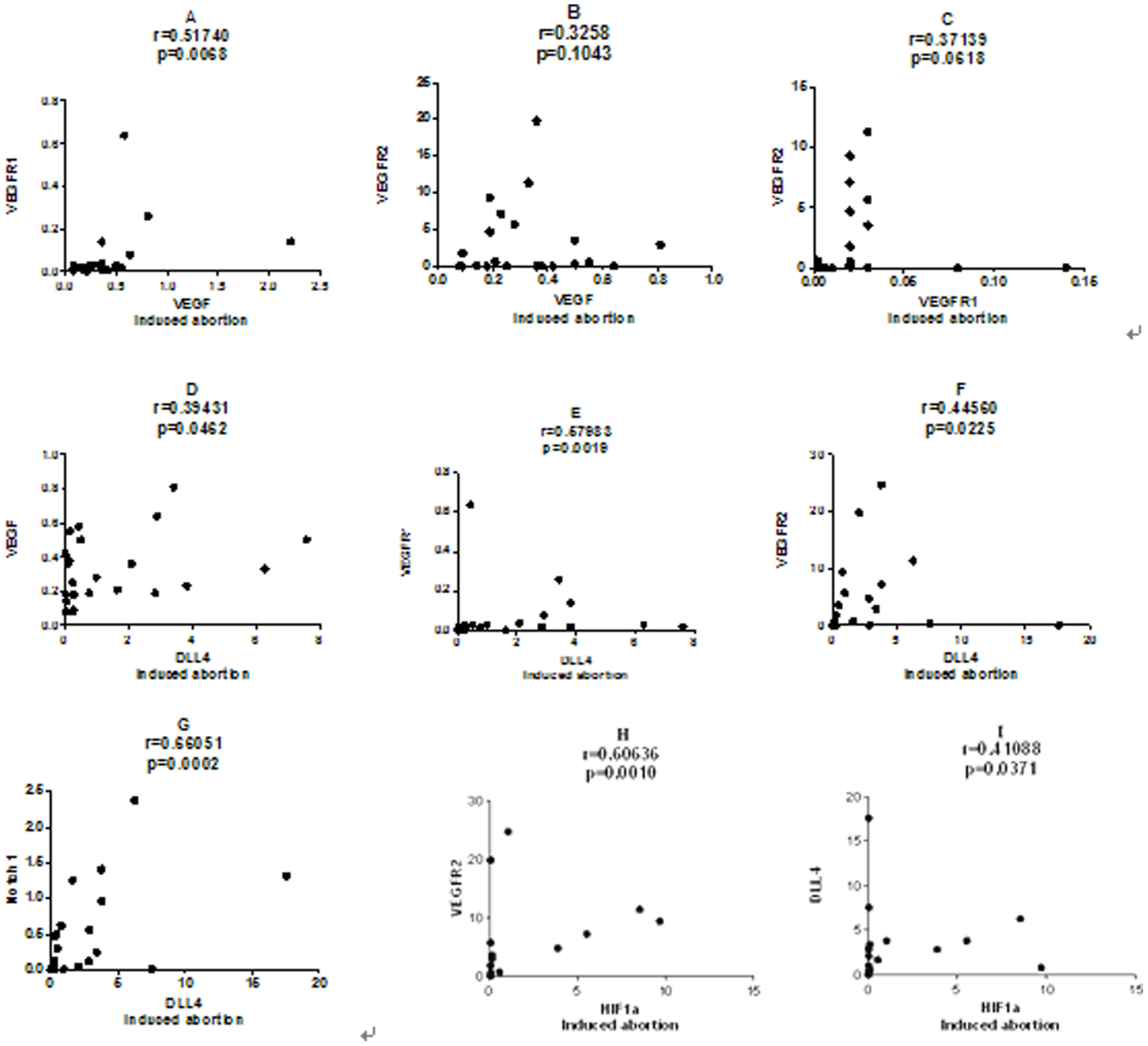
Correlation analysis in induced abortion. A.VEGF positively correlated withVEGFR1. B.VEGF expression close positively correlated with VEGFR2. C.VEGFR1 expression close positively correlated with VEGFR2. D-,G.Dll4 positively correlated with VEGF,VEGFR1, VEGFR2, Notch1. H,I. HIF-1a positively correlated with VEGFR2 or Dll4.

Spearman correlation coefficients have also been calculated between these factors in induced abortion. VEGF was positively correlated with VEGFR1 (r = 0.51740, p = 0.0068), and close positively correlated with VEGFR2 (r = 0.32586,p = 0.1043); VEGFR1 was close positively correlated with VEGFR2 (r = 0.37139,p = 0.0618); Dll4 was positively correlated with VEGF (r = 0.39431, p = 0.0462), VEGFR1 (r = 0.57983,p = 0.0019) or VEGFR2 (r = 0.44560,p = 0.0225); And Dll4 was positively correlated with Notch1 (r = 0.66051, p = 0.0002); HIF-1a was positively correlated with VEGFR2 (r = 0.60636, p = 0.0010) or Dll4 (r = 0.41088,p = 0.0371). (Fig. 4).

## Discussion

Studies have shown that a successful pregnancy depended on enough villous angiogenesis, only with this can it supply adequate oxygen and nutrients [19]–[21]. In early pregnancy, the development of trophoblast cells (before 12 weeks of pregnancy) is a hypoxic environment, and trophoblast cells are exposed to a relatively low-oxygen environment and the adaption of trophoblast cells to hypoxic environment is the key to successful pregnancy [22]. Missed abortion was associated with severe low-oxygen and less angiogenesis. In our study, we found that HIF-1a was expressed in induced abortion, and significantly up-regulated in missed abortion.

During early pregnancy, vasculogenesis is one of the essential steps in appropriate embryonic vascular system. And during the embryonic angiogenesis development, VEGF and their VEGF receptors (VEGFRs) are the heart of this signaling network [23]–[24]. The concept of VEGFR2 as the main mediator of VEGF biological effect has now been generally accepted that it plays a key role in embryonic angiogenesis. This is also confirmed by data on inhibition of angiogenesis upon inactivation of VEGFR2 [25]–[29]. And it was supposed that negative regulation of the effect of VEGF on vascular endothelial cells rather than mitotic signal transduction might be the main function of VEGFR1. In our study, in induced abortion, VEGF was positively correlated with VEGFR1 and close positively correlated with VEGFR2, and VEGFR1 was also close positively correlated with VEGFR2. Our findings indicated that this balance promotes the angiogenesis to maintenance the normal pregnancy. However, in missed abortion, the balance was interrupted. VEGF was down-regulated and VEGFR1 was elevated, while VEGFR2 was found no significant change, which may contribute to the missed abortion.

During pregnancy, with the changes in oxygen levels, placental trophoblast vascular network angiogenesis will change accordingly [30]. Hypoxia is one of the most important factors inducing VEGF expression. VEGF gene expression is up-regulated in hypoxia via the oxygen sensor HIF-1a [31]. Our study showed that HIF-1a was positively correlated with VEGFR2 in induced abortion. But in missed abortion, it was in a severe hypoxic environment, HIF-1a may decrease the expression of VEGF, then inhibit the angiogenesis [32]. The researcher Vuorela using immunohistochemical method analysis found that patients with missed abortion had less expression of VEGF in trophoblast [33]. Our study also demonstrated the expression of VEGF was statistically reduced and HIF-1a was negatively correlated with VEGF in missed abortion.

When VEGF affects endothelial is tessellated among endothelial cells in the vessel area, where activation of angiogenesis takes place, the tip-cell specific characteristics are preferably acquired by endothelia cells devoid of Notch1 expression. It appears that the signaling system Dll4/Notch plays a key role in the regulation of angiogenesis [11], [34].The mechanism of the Dll4/Notch associated signaling cascade that determines the differing behavior of endothelial cells is evidently connected with the intracellular signal transduction stimulated by VEGF [35]–[37]. In hypoxic tissue, Dll4 signaling by tip cells suppress VEGFR2 expression in neighboring stalk cells via Notch pathway activation, inhibiting tip cell phenotype and ectopic sprout formation [31]. The interplay of VEGFR2 and Notch signaling controls the angiogenic sprouting. Consistently, our study found that in induced abortion, there were positive correlations between HIF-1a, Dll4, Notch, and VEGFR2. These indicated that the low-oxygen environment, with the high expression of HIF-1a, leads to up-regulation of the ligand Dll4, while Dll4-mediated activation of Notch1 receptors in a neighboring cell activate VEGFR2 expression then promote the angiogenesis. Other Notch ligands or receptors may take part in the interaction with VEGF in the process of placental angiogenesis. However, in missed abortion, there were no correlations between Dll4 and VEGF/VEGFR1/VEGFR2, which indicated that the positive feedback between Dll4/Notch and HIF-1a-VEGF might be destroyed.

In order to confirm the interaction of Dll4/Notch and VEGF pathway, HUVECs cultured on dishes were transfected with Dll4-expressing plasmid. Compared with HUVECs transfected with GFP control plasmid, Dll4, Notch1 and VEGFR1 were up-regulated while VEGF and VEGFR2 were down-regulated in Dll4 transfected HUVECs (data not shown).

In conclusion, our findings support the hypothesis that the association of HIF-1a-VEGF and Dll4/Notch1 may have a role in missed abortion. Further functional study will be necessary to determine the mechanisms of missed abortion.
